# A Scoping Review of Audiovisual Integration Methodology: Screening for Auditory and Visual Impairment in Younger and Older Adults

**DOI:** 10.3389/fnagi.2021.772112

**Published:** 2022-01-27

**Authors:** Aysha Basharat, Archana Thayanithy, Michael Barnett-Cowan

**Affiliations:** Department of Kinesiology, University of Waterloo, Waterloo, ON, Canada

**Keywords:** aging, multisensory, integration, sensory perception, auditory acuity, visual acuity, audition, vision

## Abstract

With the rise of the aging population, many scientists studying multisensory integration have turned toward understanding how this process may change with age. This scoping review was conducted to understand and describe the scope and rigor with which researchers studying audiovisual sensory integration screen for hearing and vision impairment. A structured search in three licensed databases (Scopus, PubMed, and PsychInfo) using the key concepts of multisensory integration, audiovisual modality, and aging revealed 2,462 articles, which were screened for inclusion by two reviewers. Articles were included if they (1) tested healthy older adults (minimum mean or median age of 60) with younger adults as a comparison (mean or median age between 18 and 35), (2) measured auditory and visual integration, (3) were written in English, and (4) reported behavioral outcomes. Articles that included the following were excluded: (1) tested taste exclusively, (2) tested olfaction exclusively, (3) tested somatosensation exclusively, (4) tested emotion perception, (5) were not written in English, (6) were clinical commentaries, editorials, interviews, letters, newspaper articles, abstracts only, or non-peer reviewed literature (e.g., theses), and (7) focused on neuroimaging without a behavioral component. Data pertaining to the details of the study (e.g., country of publication, year of publication, etc.) were extracted, however, of higher importance to our research question, data pertaining to screening measures used for hearing and vision impairment (e.g., type of test used, whether hearing- and visual-aids were worn, thresholds used, etc.) were extracted, collated, and summarized. Our search revealed that only 64% of studies screened for age-abnormal hearing impairment, 51% screened for age-abnormal vision impairment, and that consistent definitions of normal or abnormal vision and hearing were not used among the studies that screened for sensory abilities. A total of 1,624 younger adults and 4,778 older participants were included in the scoping review with males composing approximately 44% and females composing 56% of the total sample and most of the data was obtained from only four countries. We recommend that studies investigating the effects of aging on multisensory integration should screen for normal vision and hearing by using the World Health Organization's (WHO) hearing loss and visual impairment cut-off scores in order to maintain consistency among other aging researchers. As mild cognitive impairment (MCI) has been defined as a “transitional” or a “transitory” stage between normal aging and dementia and because approximately 3–5% of the aging population will develop MCI each year, it is therefore important that when researchers aim to study a healthy aging population, that they appropriately screen for MCI. One of our secondary aims was to determine how often researchers were screening for cognitive impairment and the types of tests that were used to do so. Our results revealed that only 55 out of 72 studies tested for neurological and cognitive function, and only a subset used standardized tests. Additionally, among the studies that used standardized tests, the cut-off scores used were not always adequate for screening out mild cognitive impairment. An additional secondary aim of this scoping review was to determine the feasibility of whether a meta-analysis could be conducted in the future to further quantitatively evaluate the results (i.e., are the findings obtained from studies using self-reported vision and hearing impairment screening methods significantly different from those measuring vision and hearing impairment in the lab) and to assess the scope of this problem. We found that it may not be feasible to conduct a meta-analysis with the entire dataset of this scoping review. However, a meta-analysis can be conducted if stricter parameters are used (e.g., focusing on accuracy or response time data only).

**Systematic Review Registration:** https://doi.org/10.17605/OSF.IO/GTUHD.

## Introduction

The proportion of the world's population over 60 years of age is estimated to increase to approximately 2 billion individuals by 2050, nearly doubling from 12% of the world population to 22% (World Health Organization, 2018a). With such a drastic shift in global demographics, the incidence of age-related chronic health conditions is also expected to increase. Indeed, the prevalence of audition and vision degradation increases with age and can have global impacts on cognition (Salthouse et al., 1996; Baltes and Lindenberger, 1997; Porto et al., 2016) and temporal perception (Gordon-Salant and Fitzgibbons, 1999; Grose and Mamo, 2010; de Boer-Schellekens and Vroomen, 2014; Brooks et al., 2018). Combining information across the senses can improve localization, discrimination, and speed of responses to objects, however, the central nervous system (CNS) must bind together the appropriate signals (Calvert et al., 2004). One cue that the CNS can use to determine whether or not stimuli should be bound together into a single percept (multisensory integration) is the temporal relation, how close in time two or more signals are to one another; research has revealed, using not only non-human animal models but also through studies conducted with humans, that signals that appear closer in time are more likely to be integrated [Vroomen and Keetels, 2010; see also King (2005) for a review of strategies used by the CNS to bind appropriate cues]. As temporal perception is affected by changes in unisensory processing, changes in auditory and visual acuities can act as indicators that may provide insight into changes associated with the multisensory integration processes within the aging population. Within the auditory domain, an estimated 466 million people worldwide have disabling hearing loss (World Health Organization, 2021a) and it is estimated that between 25 and 40% of older adults aged 65 and over, can be classified as having hearing impairment (Yueh et al., 2003). It has been found that the prevalence of hearing loss rises with age, ranging from 40 to 66% in adults over the age of 75 years and more than 80% in those older than 85 years of age (Cooper and Gates, 1991; Yueh et al., 2003; Walling and Dickson, 2012). Further it has been found that after the age of 60, hearing typically declines by about 1 dB annually and that men usually experience greater hearing loss and earlier onset compared to women (Lee et al., 2005). More than 90% of older individuals with hearing loss have age-related sensorineural hearing loss, which is a gradual symmetric loss of hearing—predominately of higher frequencies—that is worse in noisy environments (Yueh et al., 2003). Note however, that in an epidemiology study conducted by Lin et al. (2011) where data related to hearing abilities of older adults aged 70 and over was used from the 2005–2006 cycle of the National Health and Nutritional Examination Survey, it was found that the prevalence of hearing loss varied depending on the tonal frequencies, the audiometric thresholds used to define hearing loss, and whether hearing loss was considered in the better or worse hearing ear. They reported hearing loss prevalence rates from 16.5% when hearing loss was defined as using 0.5, 1, and 2 kHz (standard pure tone averages; PTA) with a 40 dB threshold in the better ear to 99.7% when hearing loss was defined as using 3, 4, 6, and 8 kHz (high-frequency PTAs) with a 15 dB threshold in the worse ear. Although they found that most reports of hearing loss prevalence used a 25 dB threshold, standard PTA (0.5, 1, and 2 kHz) or speech frequency PTA (0.5, 1, 2, and 4 kHz), and obtained measures in either the worse or better ear, there was still a high degree of variability of hearing loss reported. The range was narrower but spanned 44.8% when using the standard PTA in the better ear to 75.1% when using speech frequency PTA in the worse ear. Thus, the definition used when measuring hearing loss is crucial especially when some researchers may be utilizing a more rigid inclusion criteria as compared to others.

Shifting our focus toward the visual domain, worldwide, approximately 185 million people over the age of 50 years are visually impaired (World Health Organization, 2017), with cataracts, age-related macular disease, and refractive errors being the most common causes of visual impairment in older adults (Buch et al., 2001; World Health Organization, 2017). Although cost effective interventions such as cataract surgery and corrective glasses have shown to be effective, only 22 and 37% of individuals living in upper-middle and high-income countries respectively have reported having an eye exam during the preceding year (World Health Organization, 2017). The World Health Organization (WHO) defines visual impairment based on the International Classification of Diseases 11 (2018b) classification in the following categories for acuities measured at a distance of 2–4 m: mild visual impairment is defined as acuity worse than 6/12–6/18, moderate visual impairment is defined as acuity worse than 6/18–6/60, and severe visual impairment is defined as visual acuity that is worse than 6/60–3/60 in both eyes. In other words, the WHO defines visual impairment as best corrected visual acuity of <20/40 but ≥20/400, while many researchers (especially in the US) commonly define visual impairment as best corrected visual acuity that is worse than 20/40 but better than 20/200 in the better eye (Buch et al., 2001; World Health Organization, 2018; also see Supplementary Table 1 for details regarding the WHO's definition of visual impairment. This table contains Snellen and LogMAR values). In a study conducted by Buch et al. (2001) comparing the prevalence of visual impairment in 944 individuals as defined by the WHO and the criteria most commonly used in the US, it was found that 2.6% of those aged 70–74 years and 4.8% of those aged 75–80 years had visual impairments according to the WHO's definition (worse than 20/60–20/400 in the better eye; World Health Organization, 2004). However, these values differed based on the criteria used in most US studies where 3.1% of those aged 70–74 years and 8.0% of those between 75 and 80 years of age had visual impairment. Here, once again, we are reminded that the definition of impairment used by researchers is crucial and that some researchers may be excluding more participants than others.

Given the changes in sensory acuities associated with aging, accounting for such changes in hearing and vision is crucial as it may increase the quality and validity of the data obtained. The integration of auditory and visual cues into a unified percept is a fundamental process with an evolutionary benefit as it allows the observer to respond to external events more quickly and accurately relative to unisensory information alone (Stein and Stanford, 2008). Such an ability to integrate auditory and visual cues into a coherent percept has been thought to be beneficial for everyday function, for example in improving perception of speech in noise (Sumby and Pollack, 1954) and in improving driving performance (Ramkhalawansingh et al., 2016), both of which are especially relevant for the aging population. Research has time and again revealed that there are three principles that underlie multisensory processing, the first two principles suggest that the more temporally and spatially coincident (Meredith et al., 1987; Stein et al., 2014; Baum and Stevenson, 2017) two sensory cues are, the more likely they are to be bound together and result in a unified percept. The third principle states that unisensory signals that are weakly effective on their own are more likely to benefit from integration (Stein et al., 2009, 2014; Baum and Stevenson, 2017). This third principle of inverse effectiveness however does not hold true when the unisensory component that would be bound into a multisensory percept becomes unreliable and can result in a reduction in multisensory benefits as observed through models of optimal integration (Ross et al., 2007; Ma et al., 2009; Baum and Stevenson, 2017). Indeed, effective multisensory integration is dependent on both peripheral sensory organs as well as higher cognitive processes. As significant changes in sensory systems (e.g., decrease in visual acuity and an increase in auditory acuity thresholds) and cognitive function (e.g., decline in executive function and memory) are associated with healthy aging, it is not surprising that multisensory integration also changes with age (Rapp and Heindel, 1994; Kalina, 1997; Liu and Yan, 2007; Mozolic et al., 2012; Baum and Stevenson, 2017; Fjell et al., 2017). Indeed, older adults have been found to have longer response times in audiovisual detection tasks (†Laurienti et al., 2006; †Mahoney et al., 2011; †Couth et al., 2018; †Basharat et al., 2019), exhibit wider temporal binding windows (TBWs; the window of time within which information from different modalities is integrated and perceived as simultaneous; †Setti et al., 2011b; †Bedard and Barnett-Cowan, 2016; †Basharat et al., 2019), are more likely to be distracted by irrelevant stimuli within and across modalities [Poliakoff et al., 2006; see de Dieuleveult et al. (2017) for a detailed review regarding the effects of aging on multisensory integration], but they are also more likely to exhibit greater multisensory enhancement [see Mozolic et al. (2012) and de Dieuleveult et al. (2017) for detailed reviews] compared to younger adults. Further, it has been found that such changes in multisensory integration are exacerbated in those living with mild cognitive impairment and dementia. Research has revealed that those living with MCI and dementia tend to have longer response times, exhibit wider temporal binding windows, are more likely to experience attention impairment, and are less likely to benefit from multisensory enhancement compared to healthy controls (Wu et al., 2012; Chan et al., 2015; Murray et al., 2018). These results suggest that both cognitive function and sensory abilities must be accounted for when conducting multisensory integration related research with the aging population.

A decline in sensory abilities can affect the reliability, or the precision of a sensory estimate, with which the central nervous system integrates cues from auditory and visual modalities and can thus reduce the benefits typically gained through the multisensory process (Ernst and Bülthoff, 2004; Odegaard and Shams, 2016). Note however, that reduced acuity may also help to explain the increased benefits of multisensory integration in the aging population through the lens of the principle of inverse effectiveness (Mozolic et al., 2012). With a decline in auditory and visual acuity, the unisensory cues from these modalities would be presented just above threshold levels, thus the principal of inverse effectiveness would predict that integration of these weakly effective cues would produce gains much larger than the sum of their parts, suggesting that individuals with reduced sensitivity or acuity (i.e., older adults) may experience enhanced sensory integration. Thus, accounting for age-related sensory loss is essential in multisensory literature as it impacts the reliability of the incoming information and thus the likelihood of integration. It should however be noted that in a recent review conducted by de Dieuleveult et al. (2017) where the performance of older adults was compared to younger adults on unisensory and multisensory stimuli, it was found that although older adults did not always exhibit slower response times on the unisensory stimuli, they continued to show multisensory facilitation, indicating that inverse effectiveness may be one of many processes involved in the enhancement observed for multisensory cues in older populations (†Peiffer et al., 2007; Guerreiro et al., 2014, 2015).

Regardless of the underlying mechanisms, some research shows that changes in audition and vision impact temporal perception, not just within each modality, but also between these sensory modalities. Within the auditory domain, older age impairs temporal order judgments (Gordon-Salant and Fitzgibbons, 1999), duration discrimination (Fitzgibbons and Gordon-Salant, 1994, 1995; Gordon-Salant and Fitzgibbons, 1999), and reduces sensitivity to temporal fine structure (Grose and Mamo, 2010). Within the visual modality, age also impairs visual temporal judgments (de Boer-Schellekens and Vroomen, 2014), reduces flicker sensitivity (Mayer et al., 1988; Kim and Mayer, 1994), and reduces critical flicker frequency (Lachenmayr et al., 1994). When assessing age-related changes to audiovisual temporal perception, researchers find that older adults are more susceptible to the sound-induced flash illusion (†Setti et al., 2011a; McGovern et al., 2014), are more susceptible to the temporal ventriloquist effect (de Boer-Schellekens and Vroomen, 2014), and have wider temporal binding windows (†Bedard and Barnett-Cowan, 2016; †Basharat et al., 2018, 2019). Further, as aging increases the prevalence of ocular disease and hearing loss, this can lead to impairment in temporal perception (Phipps et al., 2004; Gin et al., 2011; Gallun et al., 2014). Although many, but not all, audiovisual multisensory paradigms include auditory- and visual-only conditions to gain insight into the workings of auditory and visual systems, we believe that accounting and screening for age-abnormal changes in the auditory and visual modalities will allow researchers to draw more reliable conclusions related to how audiovisual integration (the binding of auditory and visual cues into a unified percept) changes with age without being confounded by uncorrected vison and hearing. Our preliminary search revealed that researchers are not employing as much scientific rigor as would be necessary to account for auditory and visual acuity changes within the multisensory integration literature. While some researchers rely on self-reported measures obtained from participants, and others measure acuities in the lab or research centers to determine eligibility, some researchers however do not collect or account for visual and/or auditory acuities whatsoever. Further, a standardized criterion for what constitutes “normal” vision and hearing does not seem to be used and does not exist within the multisensory integration literature (Brooks et al., 2018).

Here, we aimed to determine the scope of the problem and collected information regarding what practices researchers are following in the literature to screen for vision and hearing impairment. We collected descriptive statistics regarding the number of researchers who screened for auditory and visual acuities (and how they reported it), those who used self-reported measures, and finally those who did not utilize any form of acuity measurements. We also aimed to determine what cut-off scores are being used when researchers do measure the acuities within a research or laboratory setting and what types of questions are asked to obtain self-reported perceptions of auditory and visual acuities. In addition to visual and auditory acuity measures, we also assessed how researchers define healthy aging (e.g., if cognitive impairment is accounted for and if so, how it is being measured). This scoping study will help provide a map of the methods researchers are utilizing and will help determine whether or not a meta-analysis can be conducted to further understand the scope of the issue with the current dataset.

## Methods

The methods of the current study have been registered with the Open Science Framework (Basharat and Barnett-Cowan, 2020). The scoping review was conducted according to the framework proposed by Arksey and O'Malley (2005) and the suggestions that have been developed by Levac et al. (2010).

### Identifying the Research Question

We posed our research questions as follows: what is known from existing literature about the types of auditory and visual impairment screening methods that are employed in the literature on multisensory integration perception in healthy aging to screen for inclusion. Based on the results obtained in this scoping study, a recommendation of whether or not a meta-analysis can be conducted to determine if significant differences exist in the findings and or conclusions drawn in studies that used self-reported vision and hearing impairment screening methods compared to studies that measured vision and hearing impairment in the laboratory will be made. We further aimed to determine the methods used to assess and classify cognitive impairment in this literature.

### Identifying Relevant Studies

Following the Arskey and O'Malley framework (2010), this stage aimed to identify the criteria that was used to select studies for inclusion in the scoping study. Although scoping studies are designed to be broad, we chose specific criteria that would help guide the search. Relevant articles were identified in MEDLINE Pubmed (earliest records available—June 30th, 2020), MEDLINE Scopus (earliest records available—June 30th, 2020), and PsychInfo (earliest records available—June 30th, 2020). We chose these databases to ensure a comprehensive coverage of health, engineering, social sciences, and psychology journals. We believe that Pubmed comprehensively covers health related articles, Scopus acts as a complimentary multidisciplinary database that covers articles from engineering, social sciences, and health, and finally PsychInfo provides coverage of articles specifically from the psychology domain.

The key concepts used in the searches were as follows: multisensory integration, audiovisual modality, and aging (with younger adults as a comparator). The key concepts were combined using the Boolean operator AND, and the search words within each concept were combined with OR (Basharat and Barnett-Cowan, 2020). As suggested by Levac et al. (2010), the team used an iterative process to identify key search terms. Initially, AB identified key articles and created keywords for each category for this review. A research librarian was then consulted who advised on, and helped modify the search strategy for the various databases used. Once the search strategies had been finalized, articles were retrieved from each database and imported into the Mendeley reference management software. Note that if an article did not contain a combination of all the search terms (i.e., multisensory, audiovisual, and aging) in the abstract, title, or in the “keywords,” it most likely did not appear in our search results.

#### Search Strategy Used for Scopus, PubMed, and PsychInfo

##### Scopus

(TITLE-ABS-KEY (multisensory OR sensory OR crossmodal OR cross-modal OR cross-sensory OR intersensory OR multimodal OR multi-modal OR asynchrony OR temporal OR temporal-order OR “temporal window” OR integration OR “window of integration” OR “temporal binding window” OR “sound-induced flash illusion” OR “reaction time” OR “response time” OR “race model” OR simultaneity OR “redundant target”)) AND TITLE-ABS-KEY (audiovisual OR “audio-visual” OR “visual-audio” OR “auditory-visual” OR “visual-auditory”) AND TITLE-ABS-KEY (aging OR aging OR “older adult^*^” OR older OR aged OR geriatr^*^ OR gerontol^*^ OR elderly OR “older persons”); 1,368 results obtained.

##### PubMed

(multisensory[tw] OR Sensory[tw] OR crossmodal[tw] OR cross-modal[tw] OR cross-sensory[tw] OR intersensory[tw] OR multimodal[tw] OR multi-modal[tw] OR asynchrony[tw] OR temporal[tw] OR “temporal window” OR temporal-order[tw] OR integration[tw] OR “temporal binding window”[tw] OR “sound-induced flash illusion”[tw] OR “reaction time”[tw] OR “response time”[tw] OR “redundant target”[tw] OR “race model”[tw] OR simultaneity[tw] OR Reaction Time [MESH] OR Discrimination, Psychological [MESH]) AND (Audiovisual[tw] OR Audio-visual[tw] or visual-audio[tw] OR auditory-visual[tw] OR visual-auditory[tw]) AND (aging[tw] OR aging[tw] OR older[tw] OR aged[tw] OR geriatr^*^[tw] OR gerontol^*^[tw] OR elderly[tw] OR Aged [MESH] OR Aged, 80 and over [MESH] OR Geriatrics [MESH]); 790 results obtained.

##### PsychInfo

((title: (multisensory) OR title: (sensory) OR title: (crossmodal) OR title: (cross-modal) OR title: (cross-sensory) OR title: (intersensory) OR title: (multimodal) OR title: (multi-modal) OR title: (asynchrony) OR title: (temporal) OR title: (temporal-order) OR title: (“temporal window”) OR title: (integration) OR title: (“window of integration”) OR title: (“temporal binding window”) OR title: (“sound-induced flash illusion”) OR title: (“reaction time”) OR title: (“response time”) OR title: (“race model”) OR title: (simultaneity) OR title: (“redundant target”)) OR (abstract: (multisensory) OR abstract: (sensory) OR abstract: (crossmodal) OR abstract: (cross-modal) OR abstract: (cross-sensory) OR abstract: (intersensory) OR abstract: (multimodal) OR abstract: (multi-modal) OR abstract: (asynchrony) OR abstract: (temporal) OR abstract: (temporal-order) OR abstract: (“temporal window”) OR abstract: (integration) OR abstract: (“window of integration”) OR abstract: (“temporal binding window”) OR abstract: (“sound-induced flash illusion”) OR abstract: (“reaction time”) OR abstract: (“response time”) OR abstract: (“race model”) OR abstract: (simultaneity) OR abstract: (“redundant target”)) OR (Index Terms: (“Sensory Integration”) OR Index Terms: (“Intersensory Processing”) OR Index Terms: (“Reaction Time”) OR Index Terms: (“Causality”) OR Index Terms: (“Perceptual Discrimination”) OR Index Terms: (“Time Perception”) OR Index Terms: (“Temporal Order (Judgment)”))) AND Any Field: ((title: (audiovisual) OR title: (“audio-visual”) OR title: (“visual-audio”) OR title: (“auditory-visual”) OR title: (“visual-auditory”)) OR (abstract: (audiovisual) OR abstract: (“audio-visual”) OR abstract: (“visual-audio”) OR abstract: (“auditory-visual”) OR abstract: (“visual-auditory”)) OR (Index Terms: (“Audiovisual Communication”) OR Index Terms: (“Visual Perception”) OR Index Terms: (“Auditory Perception”))) AND Any Field: ((title: (aging) OR title: (aging) OR title: (“older adult^*^”) OR title: (older) OR title: (aged) OR title: (geriatr^*^) OR title: (gerontol^*^) OR title: (elderly) OR title: (“older persons”)) OR (abstract: (aging) OR abstract: (aging) OR abstract: (“older adult^*^”) OR abstract: (older) OR abstract: (aged) OR abstract: (geriatr^*^) OR abstract: (gerontol^*^) OR abstract: (elderly) OR abstract: (“older persons”)) OR (Index Terms: (Aging) OR Index Terms: (“Age Differences”) OR Index Terms: (Geriatrics) OR Index Terms: (“Individual Differences”))); 304 results obtained.

### Study Selection

Following the Arksey and O'Malley framework, studies were identified to be included in the scoping study. All articles generated from the search for each journal were imported into Mendeley where duplicates were removed. Two team members (AB and AT) read the abstracts and titles of all the articles to screen the studies for inclusion based on the following criteria: (1) healthy older adults (minimum mean or median age of 60) were tested; where “healthy” was defined as not having a neurological disease (e.g., Parkinson's disease, Alzheimer's disease, cognitive impairment, depression), (2) healthy younger adults were tested (mean or median age between 18 and 35); where healthy was defined as no current, acute, or chronic disease, (3) auditory and visual integration was measured, (4) the article was written in English, (5) the article had behavioral results. Articles that included the following were excluded: (1) tested taste exclusively, (2) tested olfaction exclusively, (3) tested somatosensation exclusively, (4) tested emotion perception, (5) were not written in English, (6) were clinical commentaries, editorials, interviews, letters, newspaper articles, abstracts only, or non-peer reviewed literature (e.g., theses), and (7) focused on neuroimaging without a behavioral component. AB and AT met every week to compare their results and to discuss any issues. Any disagreements were discussed between the two reviewers until a consensus was reached or by arbitration of a third reviewer (MBC). Once this step was complete, full articles were retrieved for further evaluation. Note here, that five studies were included that tested only older adults (3,133 participants). Since they provided meaningful information, and because the primary question relates to auditory and visual thresholds, which are more affected in the older population, we decided to make an exception for these studies and included them in this scoping review.

### Collating, Summarizing, and Reporting the Results

The results are presented below as described in the registered protocol for the current scoping review (Basharat and Barnett-Cowan, 2020). Descriptive data are presented in table format for variables of interest including but not limited to: title, author(s), year of publication, location(s); if the primary research question is addressed (i.e., if acuity was measured and if so, what the inclusion cut-off was, whether acuity was self-reported and if so, what questions were asked, or if acuity was unaccounted for); type of research article (original experimental research); description of participants (age, sex, inclusion/exclusion criteria); aim(s) of each study; methodology used [e.g., type of task used (e,g, detection response time (RT) task, simultaneity judgment (SJ), temporal order judgment (TOJ), etc.)]; outcome measures.

## Results

### Description of Studies and Participant Characteristics

**Figure 1:**
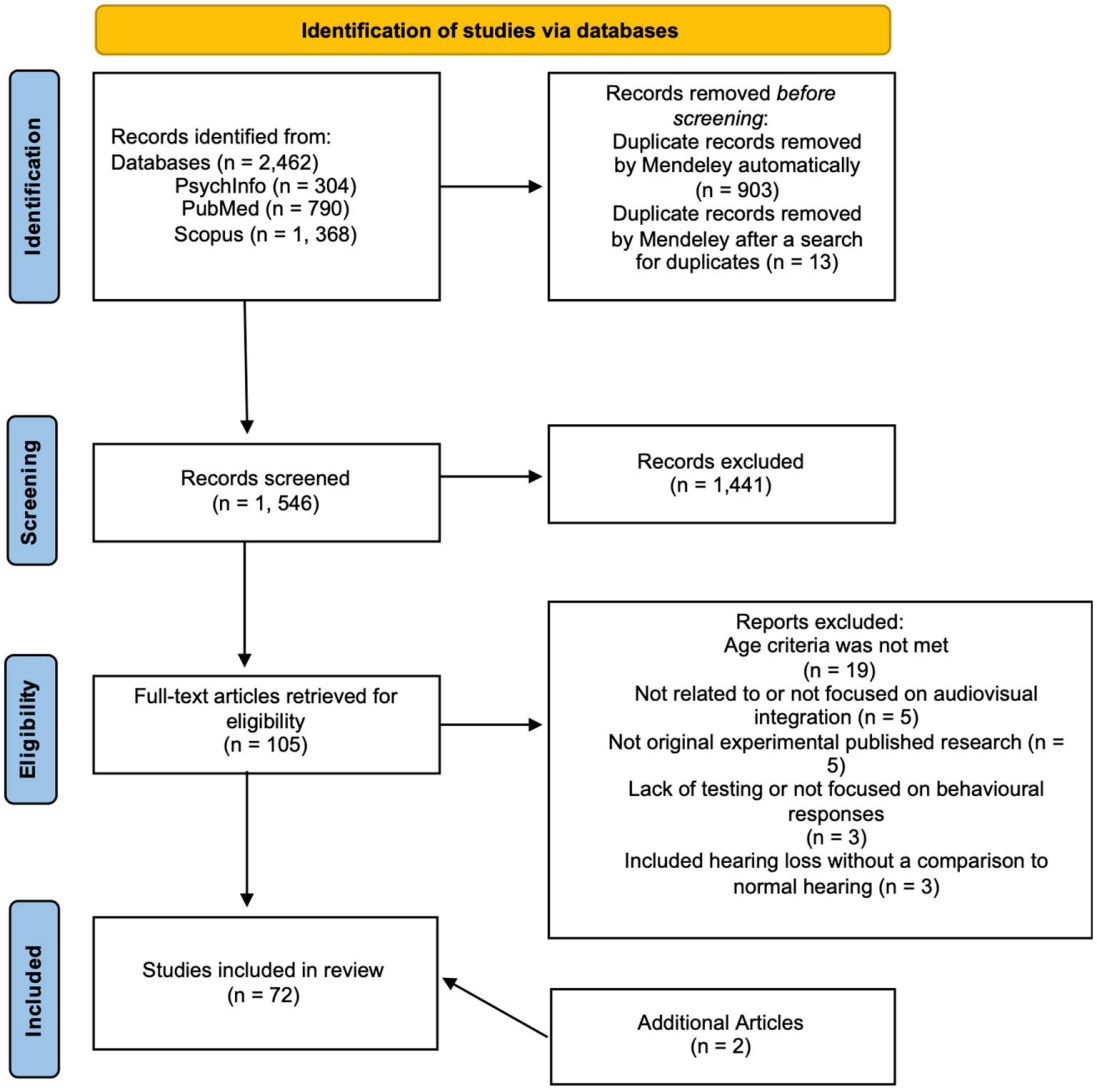
PRISMA flow diagram. This flow diagram is adopted for this scoping study from the PRISMA flow diagram for systematic review (Page et al., 2021) and includes searches of databases only.

For this scoping review, 2,462 articles were retrieved, 903 duplicates were automatically removed by Mendeley (*n* = 1,559), Mendeley was then manually checked for duplicates and 13 pairs of duplicates were found which were subsequently removed, leaving 1,546 original articles. The titles and abstracts of all 1,546 articles were reviewed. Through this process, 105 articles were selected for full article review and for further evaluation; 35 articles did not meet the inclusion criteria due to reasons spanning from age (either older adults were not included, or age was not listed), lack of relation to audiovisual integration, lack of undergoing the peer review process (thesis), or because they were reviews that did not provide sufficient information or provided information that was not relevant to this scoping study (refer to Figure 1 for further information). Note that two additional studies were included during the review process, as such, a total of 72 studies were used to assess the research questions.

**Figure 2:**
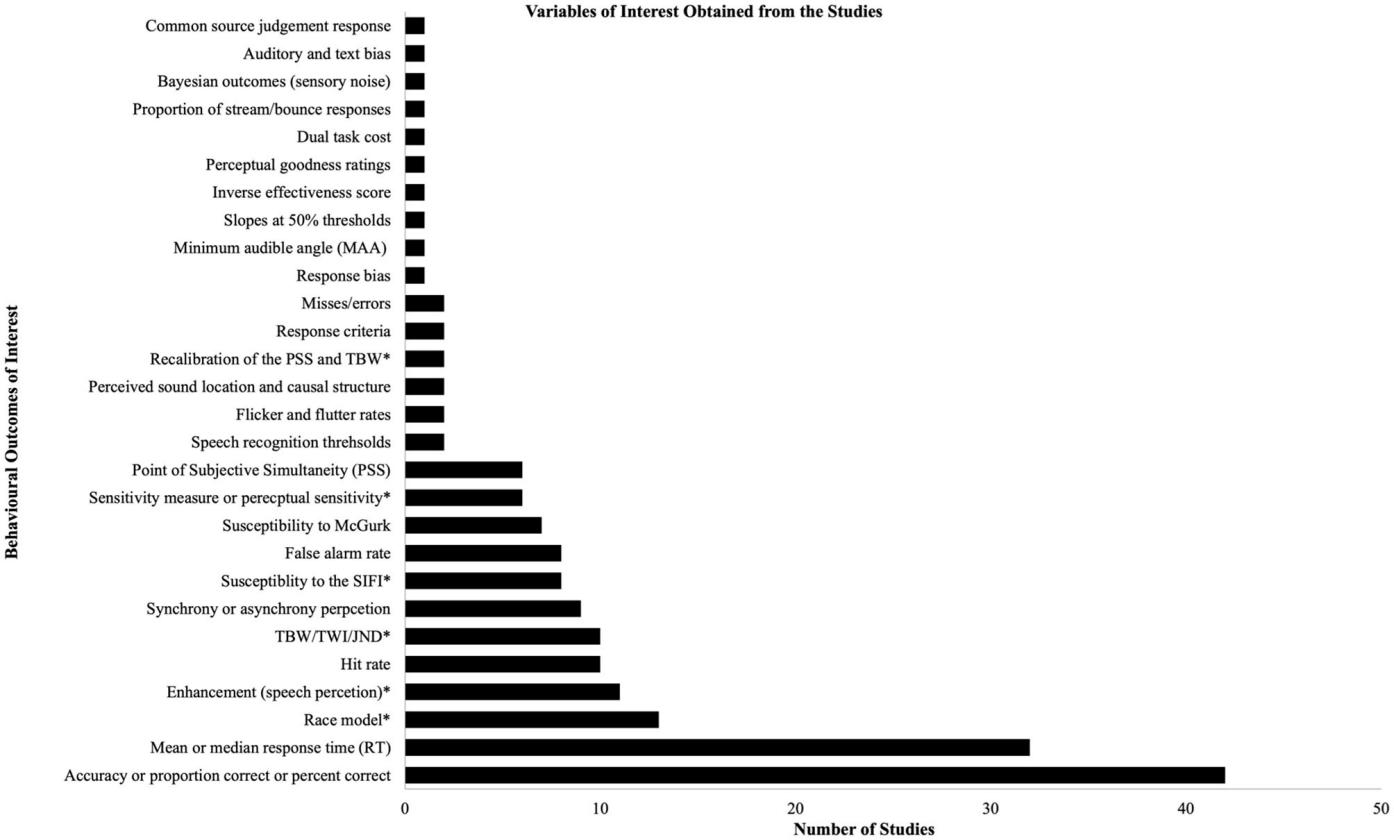
Behavioral outcomes of interest. This figure provides a visual breakdown of the behavioral outcomes of interest for the studies included in this scoping review in the form of a bar graph. Please refer to Supplementary Table 3 for further information regarding the behavioral outcomes of interest for each study as well as the type of tasks used to extract this information. Asterisks (*) in the figure indicate that further information is provided regarding their definition. Recalibration of the PSS and TBW = Recalibration of the point of subjective simultaneity and temporal binding window; sensitivity measure or perceptual sensitivity = an example is *d'*; susceptibility to the SIFI, susceptibility to the sound-induced flash illusion; TBW/TWI/JND, temporal binding window/temporal window of integration/just noticeable difference; enhancement (speech perception), auditory and/or visual enhancement for speech perception; race model, race model as a measure of enhancement (may include any or all of: cumulative distribution, difference probability, and area under the curve).

We found that the United States produced the largest number of articles (see Supplementary Tables 2, 3 for further details regarding the country of origin of the articles; note that the country of origin was determined by the affiliation of the all the authors listed on each manuscript). Various behavioral outcomes of interest were identified from the 72 studies; note that most studies extracted multiple outcomes of interest, thus the inclusion in one category does not preclude it from another category. The outcome variables of interest that were used by more than 5% of the studies are as follows: accuracy or proportion correct or percent correct (*n* = 42), mean or median response time (*n* = 32), race model as a measurement of enhancement (*n* = 13), enhancement in speech perception (*n* = 11), hit rate (*n* = 10), and the temporal binding window or temporal window of integration or the just noticeable difference (*n* = 10); see Supplementary Tables 3, 4 for further information regarding the tasks used, the aim of each study, and the outcomes of interest. See also Figure 2 for a visualization of the behavioral outcomes of interest.

As mentioned above, older adults were tested in all the articles, however, five studies did not include younger adults as a comparison. In total 6,402 participants were included where 1,624 participants consisted of younger adults while the majority of the participants (4,778) were older adults (see Tables 1, 2 for further breakdown of age and sex). Although age ranges were not included for all the studies, the majority of the studies did provide an age range for both younger and older adults (67.4% and 72.9% respectively). The following age ranges were reported for the younger group: 16–50 and 50–90 years were reported for older adults; we calculated the average range of 20.5–29.8 for younger adults and 62.0–78.7 for older adults based on the ranges provided by these studies. For studies that did not provide an age range, they provided mean ages; the following mean ages were found for younger and older adults: 22.3 and 67.8 years respectively. Many studies used normal vision (91.7%) and hearing (95.8%) as part of their inclusion criteria (measured or self-reported; see Supplementary Table 5 for further details). Further, 76.4% of studies screened for neurological or cognitive disorders (measured in lab or self-reported) and of the studies that used a cognitive assessment to account for cognitive impairment, 47.2% used the Mini-Mental State Examination (MMSE) as part of their screening protocol, while only 18.1% used the Montreal Cognitive Assessment (MoCA; see Table 3 as well as Supplementary Table 5 for a comparison of cut off scores used for inclusion in studies using the MMSE and MoCA). Further, 13.9% of studies screened for traumatic brain injury (TBI). In total 35 inclusion criteria were used (see Supplementary Table 5 for details).

**Table 1:** A breakdown of the participants included.

**Description of the studies included**	**Young males**	**Young females**	**Older males**	**Older females**
Number of participants	406	686	1,944	2,297
Percentage of sample (%)	37.18	62.82	45.84	54.16

*Note here that the percent of sample was calculated separately for young and older adults (e.g., males made approximately 37% of the sample in the younger group and approximately 46% in the older group). Further, note here that five studies were included that tested only older adults (3,133 participants) which may help to explain the large difference in numbers found between young and older adults (see Table 2 for further information). Also note that some studies did not specify the gender of their participants*.

**Table 2:** A further breakdown of the sample.

**Age group**	**Young adults (age range)**	**Older adults (age range)**	**Total males**	**Total females**
Sample size	1,624 (16–50)	4,778 (50–90)	2,350	2,983
Percentage of sample (%)	25.73	74.63	44.06	55.93

*Note, again that there are more older adults and more females compared to younger adults and males included in this scoping review. Also note that some studies did not specify the gender of their participants*.

**Table 3:** Details regarding the number of studies that used and reported the Mini Mental State Examination (MMSE) and/or Montreal Cognitive Assessment (MoCA) scores to assess cognitive function and the various scores used as part of the inclusion criteria.

**MMSE score**	**Number of studies**	**MoCA score**	**Number of studies**
> or ≥ 24	6	≥ 22	1
≥ 25	2	> or ≥ 23	3
> 26	1	≥ 24	1
> or ≥ 27	2	≥ 26	2
≥ 28	1	-	-
<2.5 SD from mean	6	-	-

*Note that some of the studies that used the MMSE and the MoCA (14 and 4 studies respectively) presented average values the participants achieved instead of a cut off score and are not reported in the table (see Supplementary Table 10 for further information). The average values for those additional studies ranged from 27.09 to 29.6 for the MMSE and 27.28 to 29 for the MoCA. Note that two studies utilizing the MMSE and two studies utilizing the MoCA did not present any results. SD, standard deviation*.

### Research Question 1: Description of Auditory and Visual Acuity Reporting

Of the 72 studies included 69 accounted for auditory acuity (i.e., measured or self-reported or both) while only 66 studies accounted for visual acuity. Of the studies investigated in this scoping review, substantially more studies both measured (46 vs. 37) and used self-reported acuity perception (41 vs. 39) to screen for auditory impairment as compared to visual impairment (see Tables 4, 5 for further information regarding how the studies included in this scoping review accounted for auditory and visual acuity). The exclusion criteria used to screen for auditory impairment were quite heterogeneous even when pure tone audiometry tests were used, with thresholds ranging from frequencies of 0.25 kHz on the lower end to 8 kHz on the higher end and intensities of 25–55 dB (see Supplementary Table 6 as well as Tables 6–8 below for details). Most studies used an auditory device (e.g., audiometer) to screen for hearing impairment, however a large majority of studies failed to report the type of test (e.g., device or custom) they used for screening eligibility. Further, only seven studies reported whether or not participants wore hearing aids, while 22 studies did not report which ear was used to screen for hearing impairment, indicating a need for improvement in reporting methods (see Table 9 below for further details). The visual modality on the other hand was slightly more homogenous, where 36.7% of the studies that measured visual acuity used the same criteria [e.g., ≥ 20/40 (6/12 or 0.3 LogMAR)] (see Supplementary Table 6 and Tables 10, 11 below for further details). Interestingly, only nine studies reported questions that were used for self-reported inclusion assessments for the auditory modality while only six studies reported the questions they used to screen for self-reported visual impairments. For the Auditory modality, these questions ranged from requiring simple “yes” or “no” responses to having more options for the participants to choose from such as “excellent,” “very good,” “good,” “fair,” and “poor.” For vision, similar questions were reported (e.g., “do you have normal or corrected to normal vision?” “yes” or “no” and “is your vision: excellent, very good, good, fair, and poor”) with an additional option of “or are you registered as legally blind” (see Supplementary Table 6 and Table 4 below for further details).

**Table 4:** Details regarding the number of studies that reported auditory and visual acuity information.

**Methods of accounting for auditory and visual acuity**	**Hearing (percentage)**	**Vision (percentage)**	**Hearing and vision (percentage)**
Acuity criteria mentioned	69 (95.83)	66 (91.66)	64 (88.88)
Acuity self-reported	41(56.94)	39 (54.16)	37 (51.39)
Studies that reported self-reported questions in the manuscript	9 (12.50)	6 (8.33)	6 (8.33)
Acuity measured objectively	46 (63.89)	37 (51.39)	33 (45.83)

*Note here, that inclusion in one category (e.g., auditory acuity measured objectively) does not exclude inclusion from a different category (e.g., auditory acuity self-reported measures)*.

**Table 5:** Details regarding the number of studies that measured auditory and visual acuities in the lab, used self-reported measures, or used a combination of both to screen for inclusion.

**Modality of interest**	**self-reported only (percentage)**	**Objectively measured only**	**Self-reported and objectively measured (percentage)**	**Measured and self-reported (percentage)**	**None (percentage)**
Hearing	23 (31.94)	26 (36.11)	20 (27.78)	46 (63.89)	3 (4.17)
Vision	29 (40.28)	25 (34.72)	12 (16.67)	37 (51.39)	6 (8.33)

**Table 6:** Details regarding the frequencies used to assess auditory acuity for inclusion.

**Frequency (kHz) used**	**Number of studies**
0.125	2
0.2/0.25	15
0.5	22
1	25
1.25	1
1.5	2
1.6	1
2	27
2.5	3
3	6
3.15	1
4	20
5	1
6	3
6.3	1
8	6

*Note that inclusion in one category does not preclude it from inclusion in another category. kHz, kilohertz*.

**Table 7:** Details regarding the thresholds used to assess auditory acuity for inclusion found in the studies included in this scoping review.

**Thresholds reported**	**Number of studies**
Hearing threshold lower than or equal to 15 dB	2
Hearing threshold lower than or equal to 20 dB	10
Hearing threshold lower than or equal to 25 dB	15
Hearing threshold lower than or equal to 30 dB	1
Hearing threshold lower than or equal to 35 dB	7
Hearing threshold lower than or equal to 40 dB	3
Hearing threshold lower than or equal to 50 dB	1
Hearing threshold lower than or equal to 55 dB	1

*Note that inclusion in one category does not preclude it from inclusion in another category. dB, decibel*.

**Table 8:** Details regarding the most commonly utilized auditory acuity inclusion criteria found in the studies included in this scoping review.

**Most common auditory acuity criteria used for inclusion**	**Number of studies**
≤ 25 dB hearing level (HL) at 0.25 – 3 kHz (both ears)	3
≤ 20 dB HL from 0.25 to 4 kHz (in both ears or not specified)	3
< or ≤ 35 dB HL at 4 kHz and < or ≤ 25 dB HL at 0.25, 0.5, 1, and 2 kHz	3
≤ 25 dB HL for 0.5, 1, 2, 4 kHz	2
0.2 – 4 kHz: no hearing loss up to 2 kHz (at ≤ 20 dB HL) and no more than mild hearing loss at 4 kHz (at ≤ 35 dB HL)	2
≤ 25 dB HL for 0.5, 1, 2 kHz in the better ear or in both ears	2

*dB, decibels; kHz, kilohertz*.

**Table 9:** Details regarding the type of test used (using a device or a custom test), whether or not participants wore hearing aids, and the ear(s) that was used to assess inclusion.

**Type of test and administration conditions**	**Number of studies**
Audiometer used to test acuity	22
Custom test used to test acuity	8
Studies that did not report the type of test they used	16
Studies where participants wore hearing aids during testing	1
Studies where participants did not wear hearing aids during testing	8
Measured in both ears	22
Measured in better ear	4
Did not report which ear was used to measure acuity	26
Studies that included a control for auditory performance	55

**Table 10:** Details regarding the criteria used to assess visual acuity for inclusion found in the studies included in this scoping review as obtained through various tests.

**Visual acuity criteria used for inclusion**	**Number of studies**
Approximately 20/20 (6/6 or 0 LogMAR)	4
≥ 20/25 (6/7.5 or 0.1 LogMAR)	8
≥ 20/30 (6/9.5 or 0.2 LogMAR)	5
≥ 20/40 (6/12 or 0.3 LogMAR)	11
≥ 20/50 (6/15 or 0.4 LogMAR)	1
≥ 20/125 (or 6/38 or 0.8 LogMAR)	1

*Note that the most commonly used criteria for exclusion was if vision was worse than: 20/40, followed by 20/25, and thirdly 20/30*.

**Table 11:** Details regarding the type of test (computerized, chart, and custom) used to test vision, the required conditions to administer this test (e.g., whether a participant used optical correction, whether binocular vision was tested, the viewing distance, etc.), if vision impairment was accounted for, and if a control condition was included for measuring only visual performance as compared to audiovisual (experimental) condition.

**Type of test and administration conditions**	**Number of studies**
Computerized test or a specialized machine used to test acuity	2
Chart used to test acuity	21
Custom test used to test acuity	4
Didn't specify the type of test used to test acuity	12
Binocular testing	10
Did not report which eye the test was conducted in	28
Near viewing distance (defined by authors as ≤ 1 m or if defined as “near” in the study)	8
Far viewing distance (defined by authors as > 1 m or if defined as “far” in the study)	11
Viewing distance not reported	25
Vision health conditions (history of cataracts, glaucoma, age-related macular degeneration, visual impairment, etc.)	17
Studies that required eye exams	2
Optical correction used (if explicitly stated)	14
Contrast sensitivity reported measured	19
Studies that included a control for visual performance	49

### Research Question 2: Can a Meta-Analysis Be Conducted?

It is quite difficult to determine whether a meta-analysis can be conducted with the articles included in this scoping review. The data reveals heterogeneity not only in tasks that were used to measure multisensory integration (e.g., target discrimination, sound localization tasks, simultaneity judgment, temporal order judgment, etc.) and the behavioral outcome of interest (e.g., accuracy/proportion correct, mean or median response time, temporal binding window, etc.) but also in how hearing and visual impairment were screened. If meta-analyses are to be used to address specific research questions, we recommend that they use specific behavioral outcomes that were most used in the literature included in this scoping review (see Supplementary Tables 3, 4 as well as Figure 2 for further information regarding the behavioral outcomes of interest. See also Supplementary Table 7 for main results). Additionally, many of the studies used unique stimuli and some did not to use control conditions, which may also impact the behavioral outcomes observed and thus should also be taken into account when thinking about conducting a meta-analysis (see Tables 9, 11 below as well as Supplementary Tables 8, 9).

## Discussion

Our review demonstrates that only 63.8 and 51.4% of studies examining audiovisual integration in aging, measure auditory thresholds and visual acuities respectively and that less than half of the studies (45.8%) that measure acuities screen both sensory modalities for age-abnormal changes. Further, a key finding is that a consistent definition of what constitutes normal hearing and vision is not employed within studies that screen for audiometric thresholds and visual acuities. Additionally, we found that although 41 and 39 studies use self-reported measures to screen for normal hearing and vision respectively, only nine studies reported the questions that were presented to participants for auditory screening, while only six studies reported the questions used to screen for self-reported visual impairment (see Tables 4, 5 below and Supplementary Table 6 for further information). In addition, as one may expect, a variety of tasks and behavioral outcomes of interest (e.g., discrimination or detection, mean response time, susceptibility to the sound induced flash illusion, etc.; see Supplementary Tables 3, 4 and Figure 2 above for details) were used in the studies selected for this scoping review; thus, the variability present in the data, from the screening measures to the multiple different tasks used makes it difficult to recommend a meta-analysis at the moment. It should however be noted that of the 2,462 articles, the 72 that were selected based on the inclusion and exclusion criteria specified above in the methods section and in the protocol for this review (https://osf.io/v3snz/; Basharat and Barnett-Cowan, 2021) were more focused on the aging process rather than on specific behavioral outcomes related to any specific task. Thus, future researchers whose research questions can be addressed using a meta-analysis can use either a rigid criterion (e.g., include studies that tested discrimination or detection response time only) to look for studies that use the same tasks or a more lenient criteria (e.g., compare the impact of aging on additional sensory modalities including somatosensation in a given task such as for detection or discrimination tasks) to capture a larger set of studies.

Our results indicate that more studies measured auditory thresholds compared to visual acuity (46 vs. 37 respectively; see Table 4 for further information). This is not surprising given that the prevalence of hearing loss rises with age ranging from 46 to 60% to more than 80% in adults aged 75 and 85 years respectively (Cooper and Gates, 1991; Yueh et al., 2003; Walling and Dickson, 2012) and is much higher than the prevalence for visual disorders that range from 2.6 to 8.0% in adults aged 70–74 and 75–80, respectively (Buch et al., 2001). However, we recommend testing both sensory modalities to ensure that stimuli presented to all participants are perceived at the appropriate thresholds (e.g., suprathreshold) required for accurate results. Note however that depending on the study design and the types of stimuli used, additional control conditions may be required. Further, and as alluded to above, the studies of audiovisual integration included in this review have adopted inconsistent screening definitions, especially for the auditory acuity, making it difficult to compare results between studies. A potential solution for this lack of standardization, is using the definitions of hearing loss and visual impairment that are recommended by the WHO; hearing loss is defined as “a speech-frequency pure-tone average at 0.5, 1, 2, and 4 kHz frequencies of >20 dB HL in both ears” and visual impairment is defined as “ <20/40 (no visual impairment) but greater than or equal to 20/400 (severe visual impairment) in both eyes” (Mathers et al., 2000; World Health Organization, 2018b, 2021b). We also found that more females than males were tested, both in the younger (62.8%) and older (54.2%) adult populations. Surprisingly, 29.2 and 34.7% of the studies failed to report gender for older and younger adults respectively, which may impact the ratio of men to women currently seen in this review. Note here that five studies were included that only tested older adults and if those studies were removed, we would be left with a comparable sample to the younger population of 461 older males and 657 older females (compared to 406 younger males and 688 younger females). However, we decided to keep these articles in the scoping review as they provide useful information regarding the screening procedures for inclusion of older adults, used in the literature.

Although a large number of studies (76.39%) used cognitive reporting to ensure that the participants included were cognitively intact (MMSE, MoCA, and DemTect, a dementia screening test, self-reported lack of cognitive impairment), many different scores were used to include or exclude individuals (see Table 3 and Supplementary Table 5 for further information regarding the inclusion criteria used for cognitive impairment and for various other inclusion criteria used by the studies included in this review). Although the variability in scores was somewhat expected as various cut off scores have been used for the detection of mild cognitive impairment (MCI) for both the MoCA (26, 25, 24, and 23; Nasreddine et al., 2005; Luis et al., 2009; Davis et al., 2015; Ciesielska et al., 2016; Milani et al., 2018) and the MMSE (28, 27, 26, 24; Anderson et al., 2007; Markwick et al., 2012; Creavin et al., 2016; Kvitting et al., 2019; Erdodi et al., 2020), we were surprised by the preferred use of the MMSE over the MoCA. As the MMSE was designed to screen for dementia at a time where the concept of MCI did not exist, the MoCA has been found to be a more sensitive test for detection and screening for early cognitive impairment compared to the MMSE (Markwick et al., 2012; Ciesielska et al., 2016). Further, research reveals that performance on the MMSE is affected by race, education, language, and gender (Tombaugh and McIntyre, 1992; Grigoletto et al., 1999; Wood et al., 2006), while the MoCA was designed as an alternative method of cognitive screening and is thought to account for the limitations that affect the MMSE [Nasreddine et al., 2005; Ciesielska et al., 2016; however please see a review by Siqueira et al. (2019) which indicates that both the MoCA and the MMSE are impacted by educational level]. Moving forward, we recommend that researchers use the MoCA to detect MCI as it was specifically designed to screen for mild cognitive impairment and it accounts for educational level differences through the addition of a point to the final score for those with <12 years of formal schooling (Nasreddine et al., 2005; Siqueira et al., 2019).

Further, we found that the 72 studies included in this scoping review used different tasks with various methodology, aims, and varying behavioral outcomes of interest (refer to Supplementary Tables 3, 4, 6, 8, 9 as well as Figure 2 above for details). Overall, the most common behavioral outcomes were thus “accuracy or proportion correct or percent correct” and “mean and median response time” measures. Given that the articles included used 28 different outcomes of interest to assess multisensory integration, it is difficult to suggest conducting a meta-analysis with the specific articles that we have used in this scoping review. However, we strongly believe that there is a sufficient amount of data available in the 1,500+ articles that were screened for this scoping review and thus suggest utilizing either a more rigid inclusion criteria (e.g., utilizing only speech recognition or response time tasks) or a broader inclusion criteria (e.g., including studies that do not mention the aging process) for those interested in conducting a meta-analysis.

This scoping review is not without its limitations. An inherent limitation of any given scoping review is that it provides breadth rather than depth on a topic (Arksey and O'Malley, 2005; Levac et al., 2010). While this scoping review provides a broad view of how studies are screening for age-abnormal sensory changes through the use of auditory and visual acuities, we are unable to determine the effectiveness of accounting for unisensory changes in multisensory integration research within this scoping review. As such, future research using meta-analyses is necessary to determine whether the results obtained from studies that screen for auditory and visual acuities differ from those that only use self-reported measures. We do however believe that providing a breath of knowledge will prove to be useful for researchers in understanding and further investigating multisensory integration within the aging population. Another limitation is that the majority of the literature in this review stems from developed countries (Economic Analysis Policy Division, 2020) and therefore it is not clear whether these findings extend to developing countries. However, it is also not clear whether the recommendations to correct the limitations associated with accounting for sensory acuities would not be applicable to the research conducted in developing nations, thus, we would extend our recommendations to developing nations unless future research indicates otherwise. Additional research with the inclusion of studies from developing nations is necessary to elucidate this matter. An additional limitation of the current study is that only studies published in English were included, limiting the review to articles that were either published in English-speaking countries, which may explain the predominance of the literature stemming from developed countries, or to those that had the funds for translation services. Finally, we conducted this scoping review using behavioral studies as we were concerned that behavioral studies may be conducted with less rigor as compared to neuroimaging studies, however, further research investigating the use of auditory and visual acuity screening methods with neuroimaging studies will not only provide insight, but is necessary to ensure standardized methods are used throughout the literature.

## Conclusion

In conclusion, we found that only approximately 64 and 51% of studies measure for age-abnormal hearing and vision respectively and that within these studies a consistent definition of what constitutes normal hearing and vision is not found. Further, we found that many studies screen for one sensory modality (audition) more than the other modality. Here, we recommend screening for both age-abnormal hearing and vision and using the World Health Organization's definitions of hearing loss and visual impairment. Further, we find that many researchers use the MMSE for MCI screening instead of the MoCA and we recommend the utilization of the latter cognitive assessment as it has been found to be more sensitive toward the detection of MCI. We found that many different tasks were used to assess audiovisual integration in younger and older adults ranging from speech recognition to the stream bounce task, thus various behavioral outcomes were obtained ranging from accuracy to stream bounce susceptibility, making it difficult to suggest conducting a meta-analysis with this particular dataset. We do however believe that a meta-analysis can be conducted with the abundant data that exists within audiovisual literature; if you wish to conduct a meta-analysis, we recommend using either a more strict or a less strict inclusion criteria depending on your research question of interest.

## Data Availability Statement

The original contributions presented in the study are included in the article/Supplementary Material, further inquiries can be directed to the corresponding author/s.

## Author Contributions

AB drafted the manuscript with input from AT and MB-C. AB and AT read the abstracts and titles of all 1,546 articles and 105 full-text articles to screen the studies for inclusion. Any disagreements were discussed between the two reviewers until a consensus was reached or by arbitration of a third reviewer MB-C. All authors contributed to study conception, development, manuscript revision, read, and approved the submitted version.

## Funding

This research was supported by an Ontario Research Fund grant and a Natural Sciences and Engineering Research Council of Canada Discovery Grant RGPIN-03977-2020 to MB-C. AB was supported by the Ontario Graduate Scholarship. University of Waterloo Interdisciplinary Trailblazer Fund to MB-C.

## Conflict of Interest

The authors declare that the research was conducted in the absence of any commercial or financial relationships that could be construed as a potential conflict of interest.

## Publisher's Note

All claims expressed in this article are solely those of the authors and do not necessarily represent those of their affiliated organizations, or those of the publisher, the editors and the reviewers. Any product that may be evaluated in this article, or claim that may be made by its manufacturer, is not guaranteed or endorsed by the publisher.
